# Predicting MiRNA-disease associations by multiple meta-paths fusion graph embedding model

**DOI:** 10.1186/s12859-020-03765-2

**Published:** 2020-10-21

**Authors:** Lei Zhang, Bailong Liu, Zhengwei Li, Xiaoyan Zhu, Zhizhen Liang, Jiyong An

**Affiliations:** 1 Engineering Research Center of Mine Digitalization of Ministry of Education, China University of Mining and Technology, Xuzhou, China; 2 School of Computer Science and Technology, China University of Mining and Technology, Xuzhou, China

**Keywords:** miRNA-disease associations, Graph embedding, Meta-path

## Abstract

**Background:**

Many studies prove that miRNAs have significant roles in diagnosing and treating complex human diseases. However, conventional biological experiments are too costly and time-consuming to identify unconfirmed miRNA-disease associations. Thus, computational models predicting unidentified miRNA-disease pairs in an efficient way are becoming promising research topics. Although existing methods have performed well to reveal unidentified miRNA-disease associations, more work is still needed to improve prediction performance.

**Results:**

In this work, we present a novel multiple meta-paths fusion graph embedding model to predict unidentified miRNA-disease associations (M2GMDA). Our method takes full advantage of the complex structure and rich semantic information of miRNA-disease interactions in a self-learning way. First, a miRNA-disease heterogeneous network was derived from verified miRNA-disease pairs, miRNA similarity and disease similarity. All meta-path instances connecting miRNAs with diseases were extracted to describe intrinsic information about miRNA-disease interactions. Then, we developed a graph embedding model to predict miRNA-disease associations. The model is composed of linear transformations of miRNAs and diseases, the means encoder of a single meta-path instance, the attention-aware encoder of meta-path type and attention-aware multiple meta-path fusion. We innovatively integrated meta-path instances, meta-path based neighbours, intermediate nodes in meta-paths and more information to strengthen the prediction in our model. In particular, distinct contributions of different meta-path instances and meta-path types were combined with attention mechanisms. The data sets and source code that support the findings of this study are available at https://github.com/dangdangzhang/M2GMDA.

**Conclusions:**

M2GMDA achieved AUCs of 0.9323 and 0.9182 in global leave-one-out cross validation and fivefold cross validation with HDMM V2.0. The results showed that our method outperforms other prediction methods. Three kinds of case studies with lung neoplasms, breast neoplasms, prostate neoplasms, pancreatic neoplasms, lymphoma and colorectal neoplasms demonstrated that 47, 50, 49, 48, 50 and 50 out of the top 50 candidate miRNAs predicted by M2GMDA were validated by biological experiments. Therefore, it further confirms the prediction performance of our method.

## Background

Micro ribonucleic acids (MiRNAs), small non-coding RNAs with 18–25 nucleotides, play crucial roles in controlling protein-encoding genes in humans [1]. Studies show that miRNAs are involved in the diagnosis, prognosis and treatment of a wide range of pathological processes, such as malignancies, cardiovascular diseases, viral infection, heart conditions, diabetes and mental disorders [2]. For example, biological experiments have shown that miR-155 acts as an oncogene in lymphoma [3]. As a consequence, it is essential to identify disease-related miRNAs. Some biological experimental approaches, such as PCR and microarrays [4], have been developed to detect miRNA-disease interactions. Nevertheless, the traditional biological experiments are limited by high costs, as they require large equipment, and are time consuming. Thus, many researchers have focused on computational methods to reveal experimentally invalidated miRNA-disease associations to compensate for the limitations of experimental methods [5, 6].

Some novel computational methods have been presented to predict miRNA-disease associations in recent years. These methods can be mainly divided into three categories: similarity-based methods, network model-based methods and machine learning-based methods. With the assumption that functionally related miRNAs are closely connected to similar diseases, diverse similarity measurements are defined in similarity-based methods. For example, Jiang et al. [7] used the first computational model, which scored with hypergeometric distributions to consider the direct neighbours in a miRNA network. This model proved to be inadequate as it disregarded the indirect neighbours. Xuan et al. [8] scored unlabelled miRNAs depending on functional similarity, miRNA family, miRNA cluster and the nearer neighbours. The local network similarity they employed restricted the prediction performance. Pasquier et al. [9] collected rich associations of miRNA-disease, miRNA-word, miRNA-family and miRNA-neighbour associations to build a miRNA vector. Chen et al. [10] incorporated within-scores and between-scores to rank the unidentified miRNA-disease pairs.

Network model-based methods first build a homogeneous or heterogeneous network based on miRNAs and diseases. Then, random walk, label propagation, sophisticated network algorithms or graph algorithms are exploited to explore the networks. For example, Shi et al. [11] conducted RWR (Random Walk with Restart) algorithm in the protein–protein network. However, the authors neglected miRNA-disease interactions. As the discovered miRNA targets were insufficient, Chen et al. [12] implemented RWR in a miRNA-miRNA network. Furthermore, Chen et al. [13] extended RWR into a disease-disease network. To explore bipartite subnetworks, Luo et al. [14] fulfilled two separate and concurrent unbalanced bi-random walks. In addition, Yu et al. [15] supplemented the virtual links with a hybrid recommendation algorithm to strengthen the networks. From the perspective of label propagation, Chen et al. [16] applied lncRNA-miRNA interactions to enrich data and performed label propagation. To reduce the sparsity of networks, Yu et al. [17] adopted matrix completion before label propagation. As well, Xie et al. [18] assessed similarity with KATZ in a bipartite network. Zhang et al. [19] developed a novel method, FLNSNLI, to predict miRNA-disease associations for the miRNAs without known associations. In addition, Yue et al. [20] reviewed graph embedding methods on biomedical networks.

Machine learning-based methods extract intrinsic features and devise efficient classification algorithms to identify miRNA-disease interactions. In an early method, Jiang et al. [21] randomly selected negative samples from unconfirmed miRNA-disease pairs and accomplished support vector machine (SVM) to perform the classification. Different from Jiang et al.’s method, Chen et al. [22] devised a semi-supervised classifier, which did not need negative instances. To address data noise and insufficiency, Liang et al. [23] defined an objective function based on L1-norm. Zhao et al. [24] integrated multiple weak classifiers with boosting to make the weak classifiers stronger. Furthermore, Chen et al. [25] chose the discriminative features according to the occurrence frequency. Moreover, both matrix decomposition [26–28] and collaborative filtering [29] were found to be powerful tools in predicting miRNA-disease associations. Motivated by the promising developments in deep learning, auto-encoder [30], node embedding [31] and SDNE (Structural Deep Network Embedding) [32] have attracted considerable attention in predicting miRNA-disease associations.

Although existing methods have performed favourably in revealing unidentified miRNA-disease associations, more work still needs to be done to improve prediction performance. On the one hand, some approaches are not applicable to new diseases that lack verified miRNAs. On the other hand, most approaches have limitations in obtaining discriminative features and intrinsic information from miRNA-disease interactions. The requirement for manual setting of the parameters makes the prediction methods suboptimal to obtain the best performance. Moreover, noise, incompleteness and insufficiency of the data provide more challenges.

Meta-paths can be applied to explore the structure information and capture the rich semantic information in heterogeneous networks [33]. Zhang et al. [34] used meta-paths to directly extract features from miRNA-disease interactions. They only considered the length information of meta-paths. Different from Zhang’s work, we extracted more information, such as meta-path instances, meta-path based neighbours, and intermediate nodes in the sequence except length. Moreover, to consider the meta-paths connecting miRNAs with diseases as global information, we developed a graph embedding model to learn the representations of miRNAs and diseases other than by extracting features directly. Therefore, we propose a novel multiple meta-paths fusion graph embedding model to predict unverified miRNA-disease associations (M2GMDA). Our method takes full advantage of the complex structure and rich semantic information in miRNA-disease interactions. In particular, all parameters are learned and do not need to be set manually after our model is created. In addition, M2GMDA is applicable to new diseases without confirmed miRNAs. The model includes linear transformations of miRNAs and diseases, the mean encoder of a single meta-path instance, the attention-aware encoder of meta-path type and the attention-aware multiple meta-paths fusion. With the power of multiple meta-paths fusion, attention mechanism and graph embedding, our method achieves superior prediction performance compared to other state-of-the-art methods. Experimental results with global leave one out cross validation (LOOCV) and fivefold cross validation show that M2GMDA had AUCs of 0.9323 and 0.9182, respectively. In addition, three kinds of case studies with lung neoplasms, breast neoplasms, prostate neoplasms, pancreatic neoplasms, lymphoma and colorectal neoplasms demonstrated that our method had reliable performances.

## Results

We first introduce the experimental approaches and evaluation criteria. Then, M2GMDA is compared with five classical prediction methods, and the experimental results are analysed. Finally, we conduct three kinds of case studies to further validate the prediction performance of our method.

### Experimental approaches and evaluation criteria

We collected 5430 experimentally supported miRNA-disease associations from HMDD V2.0 [33] to act as the data set in our prediction task. Then, we employed global LOOCV and fivefold cross validation strategies on the experimental data. Each one confirmed miRNA-disease pair was viewed as the test set, and the other pairs were regarded as the training set in global LOOCV. Meanwhile, the miRNA-disease associations from HMDD were randomly partitioned into five equal-sized groups in the fivefold cross validation. Next, four groups were taken as the training samples and the fifth one acted as the testing sample. To relieve randomness, we repeated fivefold cross validation 100 times and calculated the averaged results. We extracted all meta-paths with the length less than 4 in the experiments because we found meta-paths that were too long contributed little to improve the prediction. We set the node embedding dimension *Z* = 64. The other parameters in our model did not need to be set manually as they were all learned automatically.

To demonstrate the impact of the attention mechanism in M2GMDA, we compared M2GMDA with the attention mechanism and without the attention mechanism. Attention-aware meta-path type encoder and attention-aware fusion of multiple meta-path types were replaced by the mean encoder in M2GMDA without the attention mechanism to neglect attention weights. Similarly, to analyse the effect of the length of meta-paths, we compared the prediction performances with different length of meta-paths.

We considered area under the curve (AUC) as the criteria to assess experimental performance of different prediction methods. The receiver operating characteristics (ROC) curve was modelled by the true positive rate and the false positive rate with different thresholds.

### Comparisons with state-of-the-art methods

**Fig. 1:**
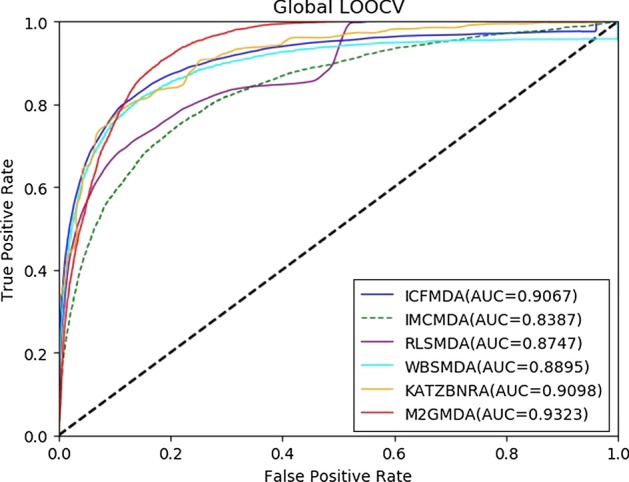
Performance comparisons of M2GMDA, IMCMDA, ICFMDA, RLSMDA, WBSMDA, and KATZBNRA in global LOOCV. As we can see M2GMDA achieved AUC of 0.9323, which was higher than the other five methods

**Fig. 2:**
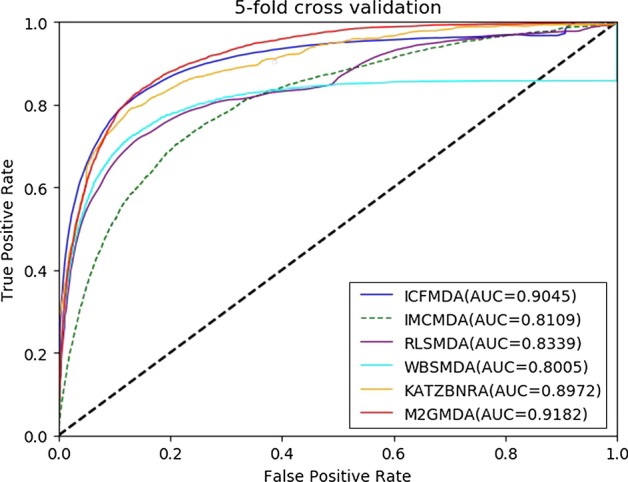
Performance comparisons of M2GMDA, IMCMDA, ICFMDA, RLSMDA, WBSMDA, and KATZBNRA in fivefold cross validation. As we can see M2GMDA achieved AUC of 0.9182, which was higher than the other five methods

To test the predictive performance of our method, we compared M2GMDA with five state-of-the-art prediction methods, IMCMDA [26], ICFMDA [29], RLSMDA [22], WBSMDA [10] and KATZBNRA [18]. The compared prediction performances of the six methods in global LOOCV and fivefold cross validation are shown in Figs. 1 and 2, respectively. Figure 1 demonstrates that M2GMDA had the highest AUC of 0.9323 in global LOOCV, indicating that it outperforms the other five prediction methods. In addition, the AUCs of IMCMDA, ICFMDA, RLSMDA, WBSMDA and KATZBNRA were 0.9067, 0.8387, 0. 8747, 0.8895 and 0.9098, respectively. Moreover, for fivefold cross validation experiments, M2GMDA also achieved the best prediction performance. The AUCs of M2GMDA, IMCMDA, ICFMDA, RLSMDA, WBSMDA and KATZBNRA were 0.9182, 0.9045, 0.8109, 0.8339, 0.8005, and 0.8972, respectively, as shown in Fig. 2. Hence, the experimental results illustrate that our method, M2GMDA, has a remarkable ability to discover the unconfirmed miRNA-disease pairs.

### Comparisons of M2GMDA with attention and without attention

**Fig. 3:**
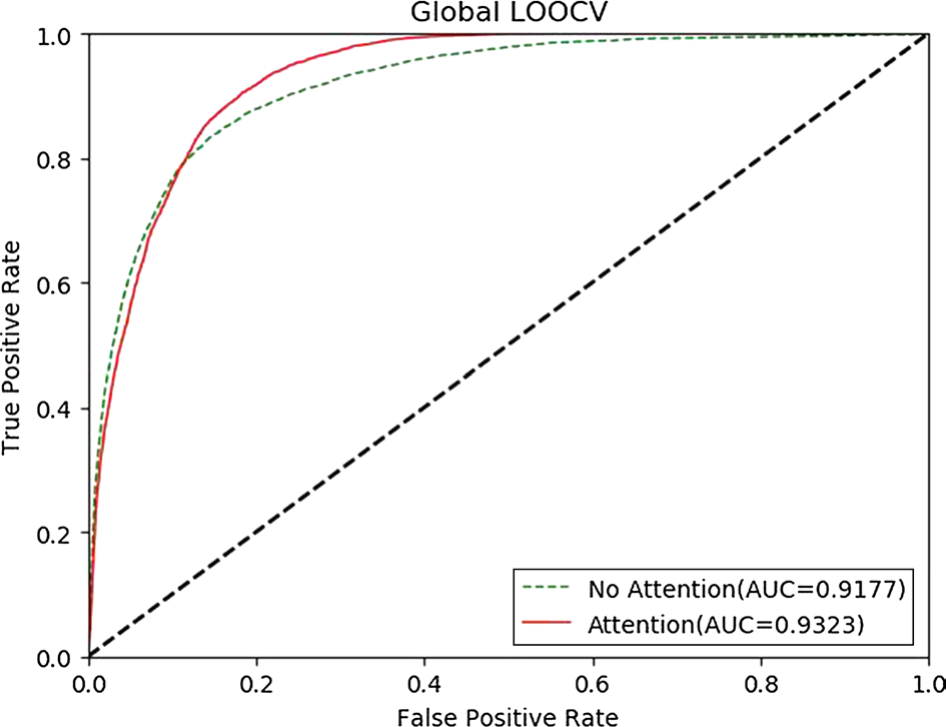
Performance comparisons of M2GMDA with attention and without attention in global LOOCV. As we can see the attention mechanism improves the prediction performance

**Fig. 4:**
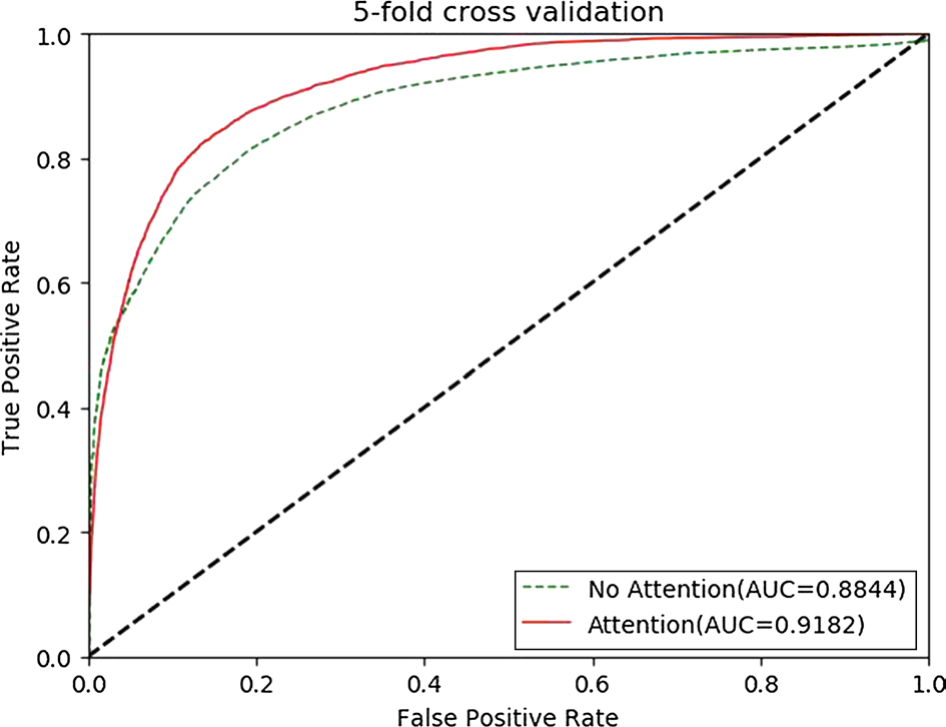
Performance comparisons of M2GMDA with attention and without attention in fivefold cross validation. As we can see the attention mechanism improves the prediction performance

We compared M2GMDA with the attention mechanism and without the attention mechanism with Global LOOCV and fivefold cross validation. The experiment results, which are shown in Figs. 3 and 4, illustrated that the attention mechanism improved the prediction performance in Global LOOCV and fivefold cross validation. The attention mechanism plays a crucial role in M2GMDA. Firstly, different nodes in a meta-path type have distinct influence in the structure information. Secondly, multiple meta-path types contribute differently to the target node. So, the attention mechanism in M2GMDA improves the prediction performance (Table 1).

**Table 1:** The top 50 miRNAs associated with lung neoplasms

miRNA	Evidence	miRNA	Evidence
hsa-mir-429	dbDEMC, PhenomiR	hsa-mir-302d	dbDEMC, PhenomiR
hsa-mir-106b	dbDEMC, PhenomiR	hsa-mir-99b	dbDEMC, PhenomiR
hsa-mir-141	dbDEMC, PhenomiR	hsa-mir-23b	dbDEMC, PhenomiR
hsa-mir-16	dbDEMC, PhenomiR	hsa-mir-383	dbDEMC, PhenomiR
hsa-mir-20b	dbDEMC, PhenomiR	hsa-mir-15b	dbDEMC, PhenomiR
hsa-mir-92b	dbDEMC, PhenomiR	hsa-mir-130b	dbDEMC, PhenomiR
hsa-mir-339	dbDEMC, PhenomiR	hsa-mir-196b	dbDEMC, PhenomiR
hsa-mir-151	PhenomiR	hsa-mir-28	dbDEMC, PhenomiR
hsa-mir-194	dbDEMC, PhenomiR	hsa-mir-299	PhenomiR
hsa-mir-302b	dbDEMC, PhenomiR	hsa-mir-10a	dbDEMC
hsa-mir-302c	dbDEMC, PhenomiR	hsa-mir-193b	dbDEMC
hsa-mir-367	dbDEMC, PhenomiR	hsa-mir-452	dbDEMC, PhenomiR
hsa-mir-215	dbDEMC, PhenomiR	hsa-mir-491	dbDEMC
hsa-mir-296	PhenomiR	hsa-mir-340	dbDEMC, PhenomiR
hsa-mir-302a	dbDEMC, PhenomiR	hsa-mir-424	dbDEMC, PhenomiR
hsa-mir-520b	dbDEMC	hsa-mir-129	dbDEMC, PhenomiR
hsa-mir-373	dbDEMC, PhenomiR	hsa-mir-516b	dbDEMC
hsa-mir-451	dbDEMC, PhenomiR	hsa-mir-449b	dbDEMC, PhenomiR
hsa-mir-195	dbDEMC, PhenomiR	hsa-mir-516a	Unconfirmed
hsa-mir-15a	dbDEMC, PhenomiR	hsa-mir-449a	dbDEMC, PhenomiR
hsa-mir-130a	dbDEMC, PhenomiR	hsa-mir-139	dbDEMC, PhenomiR
hsa-mir-372	dbDEMC, PhenomiR	hsa-mir-181d	dbDEMC, PhenomiR
hsa-mir-204	dbDEMC, PhenomiR	hsa-mir-520c	Unconfirmed
hsa-mir-153	dbDEMC, PhenomiR	hsa-mir-510	Unconfirmed
hsa-mir-488	dbDEMC, PhenomiR	hsa-mir-663	dbDEMC

### Comparisons of M2GMDA with different meta-path length

Meta-path length is an important parameter in M2GMDA. Different values of the parameter lead to different semantic scales. We compared the experiment results with different meta-path length in Global LOOCV and fivefold cross validation.

**Fig. 5:**
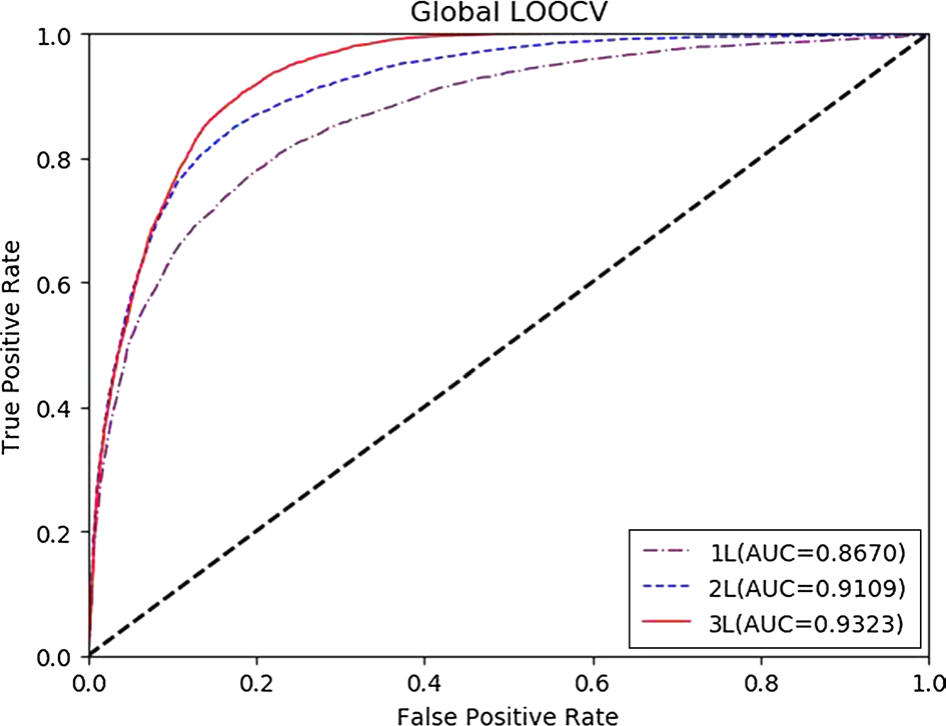
Performance comparisons of M2GMDA with different meta-path length in global LOOCV. As we can see prediction performance gets better with increase of meta-path length

**Fig. 6:**
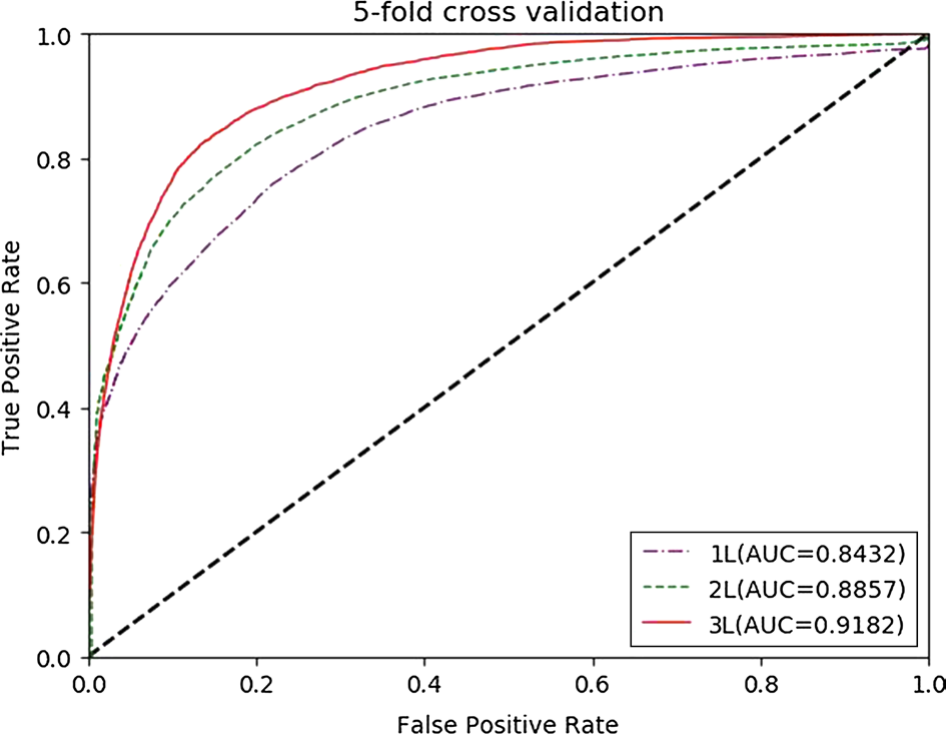
Performance comparisons of M2GMDA with different meta-path length in fivefold cross validation. As we can see prediction performance get better with increase of meta-path length

Performance comparisons are depicted in Figs. 5 and 6. We can conclude that the prediction performance gets better with increase of meta-path length. More relative node and paths are involved to model the target node as the length of meta-path increases. So, the model can aggregate more long-term dependencies between nodes. From Figs. 5 and 6, it can be seen that, with the length of meta-path increases, the number of meta-path and the time cost in generating all meta-paths increase exponentially, but the growth of prediction performance of M2GMDA slows obviously. This is due to the longer a meta-path is, the more repeatable information in shorter meta-paths it contains, which has little contribution to increasing the performance. For example, generating all meta-paths and model training may spend for 1–2 days with meta-path length of 2L, while it may spend for about one week when the meta-path length is 3. When the max meta-path length is up to 4L, the time cost may be up to weeks while the performance grows slightly. Hence, in our cases studies below, we used 3L as the max meta-path length.

### Cases studies

We implemented three kinds of case studies to further verify the prediction capability of our method to uncover miRNA-disease associations. For the first case study, we used M2GMDA to find the related unconfirmed miRNAs associated with breast neoplasms and lung neoplasms with HDMM V2.0 [35] as the data set. Then, the identified candidate miRNAs were compared to two public data sets, dbDEMC [36] and PhenomiR [37] to verify their correctness.

Lung neoplasms are devastating deadly tumours that cause a large number of deaths in both men and women worldwide [38]. It is important to diagnose lung neoplasms as early as possible because of the low 5-year survival. MiRNAs have become a promising tool in the diagnosis and treatment of lung neoplasms [39]. For example, increased miR-211 levels have been associated with increased mortality in patients with none the top 25 related miRNAs, and the third column contains the top 26–50. For the top 50 related miRNAs, 47 were confirmed to be associated with lung neoplasms by biological experimental results from dbDEMC and PhenomiR. Only 3 miRNAs were unconfirmed. For example, hsa-mir-106b, which ranks 2nd in our prediction results, has been demonstrated to promote proliferation in non-small cell neoplasms [40]. Thus, the predicted results of M2GMDA provide a novel viewport for lung neoplasms.

Breast neoplasms are common diseases with high mortality in women worldwide. It has been reported that the number of breast neoplasm patients will pass three million by the middle of the twenty-first century [41]. Medical experiments have proven that miR-142-3p is associated with breast neoplasms. We apply M2GMDA to identify the associated miRNAs for breast neoplasms and selected the top 50 candidates, which are listed in Table 2. The results showed that all the top 50 miRNAs were validated by dbDEMC and PhenomiR. In the prediction results, hsa-mir-92b, which ranked 1st, has been demonstrated to reduce the viability of breast neoplasm cells [40]. Therefore, these findings show that our prediction model provides novel evidence for studies of breast neoplasms.

**Table 2:** The top 50 miRNAs associated with breast neoplasms

miRNA	Evidence	miRNA	Evidence
hsa-mir-92b	dbDEMC	hsa-mir-181d	dbDEMC, PhenomiR
hsa-mir-30e	PhenomiR	hsa-mir-663	dbDEMC, PhenomiR
hsa-mir-151	dbDEMC, PhenomiR	hsa-mir-382	dbDEMC
hsa-mir-106a	dbDEMC, PhenomiR	hsa-mir-575	dbDEMC
hsa-mir-451	dbDEMC, PhenomiR	hsa-mir-181c	dbDEMC
hsa-mir-192	dbDEMC, PhenomiR	hsa-mir-455	PhenomiR
hsa-mir-98	dbDEMC, PhenomiR	hsa-mir-484	dbDEMC, PhenomiR
hsa-mir-32	dbDEMC, PhenomiR	hsa-mir-494	dbDEMC, PhenomiR
hsa-mir-130a	dbDEMC, PhenomiR	hsa-mir-99a	dbDEMC, PhenomiR
hsa-mir-372	dbDEMC, PhenomiR	hsa-mir-376a	dbDEMC, PhenomiR
hsa-mir-150	dbDEMC, PhenomiR	hsa-mir-154	dbDEMC, PhenomiR
hsa-mir-99b	dbDEMC, PhenomiR	hsa-mir-211	dbDEMC, PhenomiR
hsa-mir-95	dbDEMC, PhenomiR	hsa-mir-658	dbDEMC
hsa-mir-142	PhenomiR	hsa-mir-660	dbDEMC
hsa-mir-15b	dbDEMC, PhenomiR	hsa-mir-381	dbDEMC, PhenomiR
hsa-mir-130b	dbDEMC, PhenomiR	hsa-mir-432	dbDEMC, PhenomiR
hsa-mir-196b	dbDEMC, PhenomiR	hsa-mir-216a	dbDEMC, PhenomiR
hsa-mir-28	dbDEMC, PhenomiR	hsa-mir-33b	dbDEMC
hsa-mir-198	dbDEMC, PhenomiR	hsa-mir-216b	dbDEMC
hsa-mir-186	dbDEMC, PhenomiR	hsa-mir-363	dbDEMC, PhenomiR
hsa-mir-491	PhenomiR	hsa-mir-33a	dbDEMC, PhenomiR
hsa-mir-424	dbDEMC, PhenomiR	hsa-mir-454	dbDEMC, PhenomiR
hsa-mir-212	dbDEMC, PhenomiR	hsa-mir-376b	dbDEMC
hsa-mir-449b	dbDEMC	hsa-mir-217	dbDEMC, PhenomiR
hsa-mir-449a	dbDEMC, PhenomiR	hsa-mir-144	dbDEMC, PhenomiR

Then, we performed the second kind of case study to test whether our method is applicable to new diseases without experimentally supported miRNAs. Firstly, we choose prostate neoplasms for this case, as this is the most common cancer in men in the world. There are more than 100,000 men that die from prostate neoplasms in Europe alone in 2018 [43]. In this case study, we first set all miRNA-disease associations related to prostate neoplasms from HMDD 2.0 to zero. Then, M2GMDA was performed to identify the associated miRNAs for prostate neoplasms. The results shown in Additional file 1: Table S1 indicate that all the top 50 predicted miRNAs were also included in dbDEMC and PhenomiR. Secondly, to evaluate more new diseases further, we conducted the study on pancreatic neoplasms, lymphoma, lung neoplasms, colorectal neoplasms and breast neoplasms. The results of the case study of pancreatic neoplasms are listed in Additional file 1: Table S2. All of the top 50 miRNAs were confirmed by HMDD 3.2, dbDEMC and PhenomiR. At the same time, we summarize the case results for more new diseases (lymphoma, lung neoplasms, colorectal neoplasms and breast neoplasms) in Additional file 1: Table S3. For colorectal neoplasms, the found top 50 miRNAs were all confirmed. For lymphoma, lung neoplasms and breast neoplasms, only 2, 1, 1 of the top 50 miRNAs were not validated, respectively. Hence, the case study indicates that M2GMDA is applicable to new diseases.

Finally, in the third case study, we wanted to test whether M2GMDA trained with data from an older version of HMMD could identify new imported miRNA-disease pairs in a new version of HMDD. We used HMDD 3.2, dbDEMC and PhenomiR to confirm the obtained results. The results of the case study of colorectal neoplasms are listed in Additional file 1: Table S4. All of the top 50 miRNAs were confirmed by HMDD 3.2, dbDEMC and PhenomiR.

Based on the results of the three kinds of case studies, we can conclude that our prediction method is valid in predicting unconfirmed miRNA-disease associations.

## Discussion

Experimental results compared with the state-of-the-art miRNA-disease prediction methods in global LOOCV and fivefold cross validation demonstrated that M2GMDA performed better than the other prediction methods. We analysed the impact of the attention mechanism and length of meta-path. Furthermore, three kinds of case studies based on four diseases also confirmed the prediction performance of our method. The success of M2GMDA stems from three reasons. First, all meta-path instances in the miRNA-disease heterogeneous network are obtained to capture the complex relationships of miRNAs and diseases. Second, a novel meta-path instance encoder was devised to integrate the information on nodes and edges from each meta-path instance. Then, graph attention was incorporated to weight sum the different meta-path instances according to their distinction. Third, multiple meta-paths were fused to aggregate intrinsic information in multiple meta-paths. In summary, M2GMDA achieves excellent prediction by taking full advantage of the complex structure and semantic information in miRNA-disease heterogeneous network. To promote miRNA-disease prediction, we share our prediction results and provide search service on our website (https://132.232.17.50:8080/M2GMDA.jsp).

## Conclusion

To take full advantage of the complex structure and rich semantic information in miRNA-disease heterogeneous network, we present a novel multiple meta-paths fusion graph embedding model to predict unconfirmed miRNA-disease associations (M2GMDA). To enrich the information in every meta-path instance, we take into account intermediate nodes in the sequence. Attention mechanism is integrated into the meta-path encoder to distinguish different meta-path instances. Multiple meta-paths are fused according to their different contributions. Finally, the loss function is defined to train the model and obtain the learned miRNA-disease associations. Experimental results with global LOOCV and fivefold cross validation showed that M2GMDA performed better than the other state-of-the-art prediction methods. In addition, case studies show that our method achieves reliable prediction performance. In the future, we plan to explore more information in heterogeneous network to predict miRNA-disease associations more accurately. In conclusion, M2GMDA is a powerful method to identify miRNA-disease associations. To promote the research on predicting miRNA-disease associations, we published our source code and developed a web service to share our prediction results.

## Methods

**Fig. 7:**
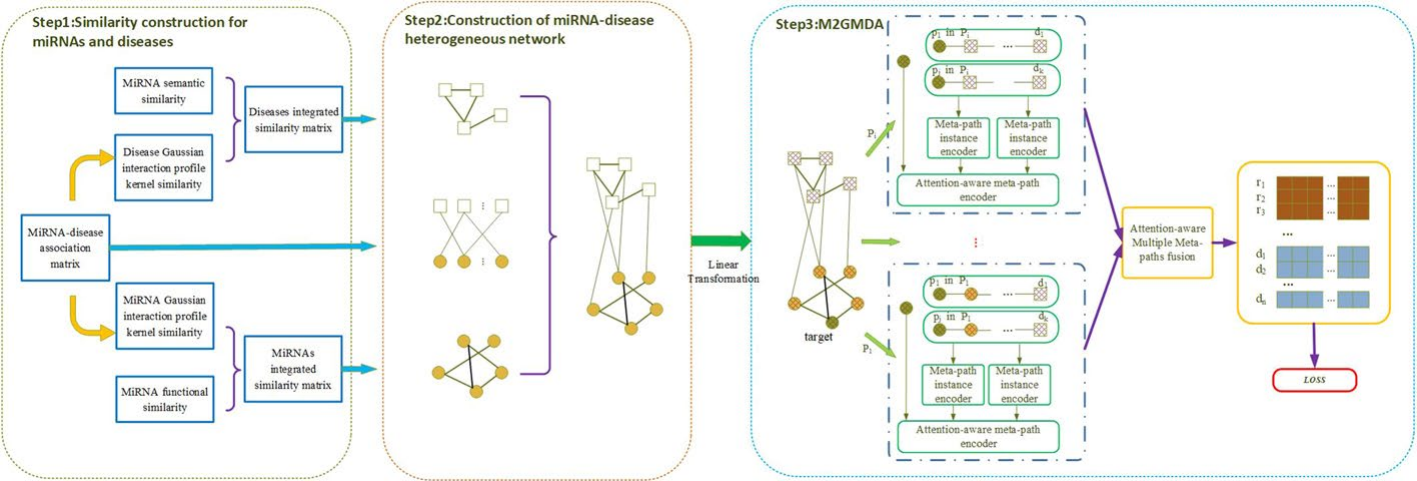
Flow chart of M2GMDA. First, miRNA integrated similarity and disease integrated similarity were calculated according to multiple measurements. Then, miRNA-disease heterogeneous network was constructed. Finally, a novel graph embedding model was used to predict the unconfirmed miRNA-disease associations

The framework for predicting miRNA-disease associations by M2GMDA is displayed in Fig. 7. First, multiple similarity measurements were adopted to calculate miRNA integrated similarity and disease integrated similarity. Second, we built a miRNA-disease heterogeneous network from experimentally confirmed miRNA-disease associations, miRNA integrated similarity and disease integrated similarity. Third, we developed a novel graph embedding model to fuse all meta-path instances to predict the unconfirmed miRNA-disease associations. The model consists of linear transformations of miRNAs and diseases, the means encoder of a single meta-path instance, the attention-aware encoder of meta-path type and the attention-aware multiple meta-paths fusion. In our model, the original features of miRNAs and diseases with various dimensions were transformed into unified latent spaces with the same dimension. Then, the means encoder of a single meta-path instance was employed to explore the sequence information of a single meta-path instance. We obtained the final representations of miRNAs and diseases by attention-aware meta-path type encoder and attention-aware fusion of multiple meta-path types. Finally, we defined the loss function to learn the parameters and predict the miRNA-disease associations.

### Construction of a MiRNA-disease heterogeneous network

#### MiRNA-disease interaction network construction

HMDD V2.0 is a popular database that consists of experimentally supported miRNA-disease interactions. We downloaded HMDD V2.0 and used it as the standard data set. For convenience, we utilized the adjacency matrix $A\in {R}^{m\times n}$ to formalize the experimentally supported interactions between miRNAs and diseases. Here, $m$ and $n$ are the numbers of miRNAs and diseases, respectively. In the matrix $A$, the element ${A}_{ij}$ equaling to 1 means that miRNA ${r}_{i}$ is related to disease ${d}_{j}$, otherwise, ${A}_{ij}$ equals to 0. In this paper, we adopted HMDD V2.0 to build $A$. There are 5430 associations between 495 miRNAs and 383 diseases in HMDD V2.0. Thus, $m=495$ and $n=383$. Therefore, we utilized $A$ to build a miRNA-disease interaction network.

#### MiRNA similarity network construction

We determined miRNA integrated similarity by combining miRNA functional similarity with Gaussian interaction profile kernel similarity as follows:

1 $$SM\left({r}_{i},{r}_{j}\right)=\left\{\begin{array}{ll} {FS}_{ij}& \quad {r}_{i},{r}_{j}\, has \,functional \,similarity\\ GM\left({r}_{i},{r}_{j}\right)& \quad otherwise\end{array}\right.$$

here ${FS}_{ij}$ stands for functional similarity of miRNAs ${r}_{i}$ and ${r}_{j}$, $GM\left({r}_{i},{r}_{j}\right)$ stands for Gaussian interaction profile kernel similarity of miRNAs ${r}_{i}$ and ${r}_{j}$.

Wang et al. defined miRNA functional similarity based on the notion that miRNAs with higher functional similarity are more likely to correlate with similar diseases [42]. Based on their work, we downloaded the functional similarity data.

In addition, Chen et al. measured the Gaussian interaction profile kernel similarity of miRNAs as follows [24]:

2 $$GM\left({r}_{i},{r}_{j}\right)=\mathrm{exp}\left(-{\alpha }_{r}{\Vert IV\left({r}_{i}\right)-IV\left({r}_{j}\right)\Vert }^{2}\right)$$

here $IV({r}_{i})$ and $IV\left({r}_{i}\right)$ indicate the *i*-th and *j*-th row of adjacency matrix $A$, respectively.${\alpha }_{r}$ is the kernel bandwidth parameter which can be formed as follows:

3 $${\alpha }_{r}=\frac{{\alpha }_{r0}}{\frac{1}{m}\sum_{i=1}^{m}{\Vert IV\left({r}_{i}\right)\Vert }^{2}}$$

here ${\alpha }_{r0}$ is the initial kernel bandwidth, which is set to 1. Thus, we can model a miRNA similarity network from miRNA integrated similarity.

#### Disease similarity network construction

We calculated the integrated similarity between two diseases based on combined disease semantic similarity and Gaussian interaction profile kernel similarity as follows:

4 $$SD\left({d}_{i},{d}_{j}\right)=\left\{\begin{array}{llc}SS\left({d}_{i},{d}_{j}\right)& \quad has \,combined \,sematic \,similarity\\ GD\left({d}_{i},{d}_{j}\right)& \quad otherwise\end{array}\right.$$

here $SS\left({d}_{i},{d}_{j}\right)$ represents the disease combined semantic similarity of diseases ${d}_{i}$ and ${d}_{j}$. $GD\left({d}_{i},{d}_{j}\right)$ represents the disease Gaussian interaction profile kernel similarity.

Disease combined semantic similarity is derived from two semantic similarity measurements of two diseases. On the one hand, Wang et al. define disease semantic similarity based on MeSH [44]. First, they define the contribution of disease $d$ in Directed Acyclic Graph ($DAG(D)$) as follows:

5 $${D1}_{D}\left(d\right)=\left\{\begin{array}{ll}1& \quad if\, d=D\\ \mathrm{max}\{\Delta *{D1}_{D}({d}^{{\prime}})|{d}^{{\prime}}\in \,children\, of\, d\}& \quad if\, d\ne D\end{array}\right.$$

here $\Delta$ is the semantic contribution delay factor.

Then, the semantic value of $D$ is obtained as follows:

6 $$DV1\left(D\right)={\sum }_{d\in T\left(D\right)}{D1}_{D}\left(d\right)$$

here $T(D)$ is the set containing $D$ and all its ancestor nodes.

Finally, they provide the similarity score of disease ${d}_{i}$ and disease ${d}_{j}$ as follows:

7 $$SS1\left({d}_{i},{d}_{j}\right)=\frac{{\sum }_{d\in T\left({d}_{i}\right)\cap T\left({d}_{j}\right)}\left({D1}_{{d}_{i}}\left(d\right)+{D1}_{{d}_{j}}\left(d\right)\right)}{DV1\left({d}_{i}\right)+DV1\left({d}_{j}\right)}$$

On the other hand, we performed another similarity analysis of two diseases as defined by Xuan et al. [8] to calculate the other semantic similarity. Xuan et al. measure semantic similarity of two diseases based on the notion that some specific diseases may have higher contributions to disease $D$. They define the contribution of $d$ in DAG as follows:

8 $${D2}_{D}\left(d\right)=-log\frac{the\, number \,of\, DAGs \,inluding \,d }{the \,numbuer\, of\, diseases}$$

Then, they measure semantic similarity $SS2\left({d}_{i},{d}_{j}\right)$ between ${d}_{i}$ and ${d}_{j}$ as the percentage of their own contributions and those of their common ancestor nodes as follows:

9 $$SS2\left({d}_{i},{d}_{j}\right)=\frac{{\sum }_{d\in T\left({d}_{i}\right)\cap T\left({d}_{j}\right)}\left({D2}_{{d}_{i}}\left(d\right)+{D2}_{{d}_{j}}\left(d\right)\right)}{DV2\left({d}_{i}\right)+DV2\left({d}_{j}\right)}$$

Here, $DV2\left({d}_{i}\right)$ and $DV2\left({d}_{j}\right)$ are defined similar to Formula (6).

Finally, we considered the average value of two semantic similarities from Wang et al. and Xuan et al. as the combined semantic similarity as follows:

10 $$SS\left({d}_{i},{d}_{j}\right)=\frac{ SS1\left({d}_{i},{d}_{j}\right)+ SS2\left({d}_{i},{d}_{j}\right)}{2}$$

Therefore, we modelled disease similarity network based on disease integrated similarity.

Finally, we integrated the miRNA-disease interaction network, miRNA similarity network, and disease similarity network to form a miRNA-disease heterogeneous network. The miRNA-disease heterogeneous network is defined as an undirected graph *G* = *(V, E)* over miRNAs ($M$) and diseases ($D$). *V* stands for node set, which consists of miRNAs and diseases. *E* stands for an edge set including three edge types, i.e., $M\to D$ or $D\to M$ indicates that a miRNA is related to a disease, $M\to M$ shows that two miRNAs are similar, $D\to D$ demonstrates that there is an edge between two diseases.

### Meta-path instances extraction from the MiRNA-disease heterogeneous network

A miRNA may be connected with a disease by one or multiple paths in the miRNA-disease heterogeneous network. The indirect and composite connections of miRNA-disease, named meta-paths, signal rich semantic information and help to understand the complex structure and semantic information of miRNA-disease interactions. Meta-paths have various types because of the differences in nodes and edges in their sequences. For convenience, we explain meta-path type, meta-path instance and meta-path based neighbour below.

**Fig. 8:**
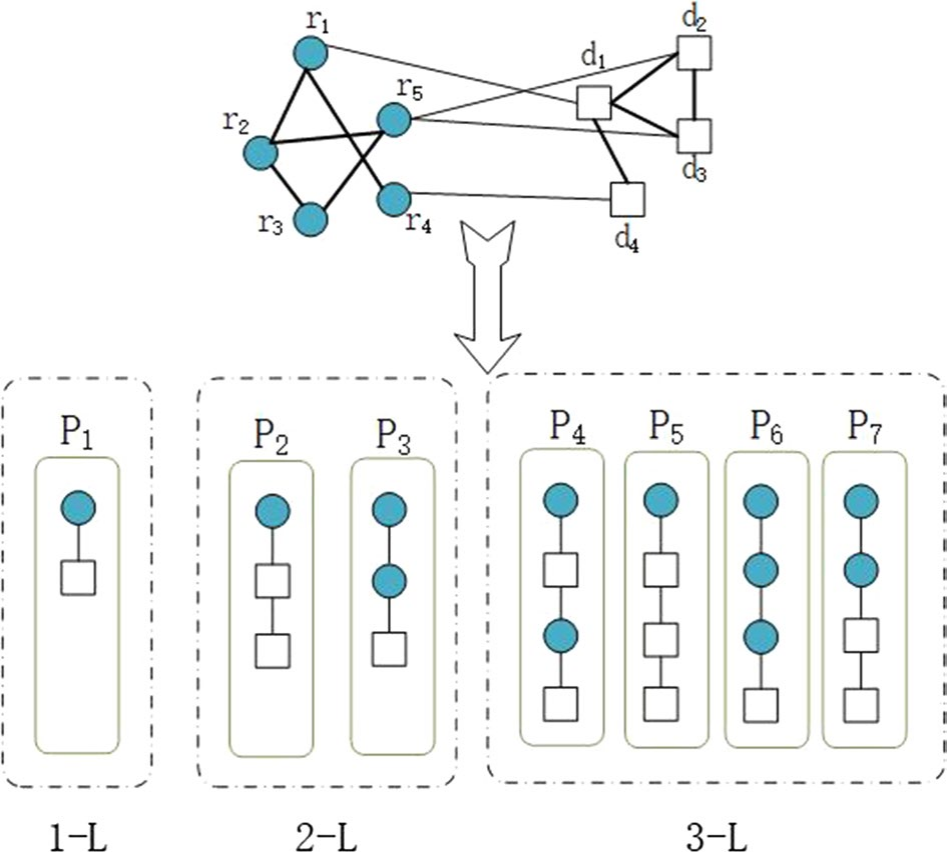
Example of meta-paths with different Lengths. Many meta-path instances were extracted from miRNA-disease heterogeneous network

First, we define meta-path type $P$ with *L-Length* as a sequence in the form of ${T}_{1}\stackrel{{R}_{1}}{\to }{T}_{2}\stackrel{{R}_{2}}{\to }\cdots {T}_{i}\stackrel{{R}_{i}}{\to }\cdots {\stackrel{{R}_{L}}{\to }T}_{L+1}$. Here,${T}_{i}\in \{M,D\}$, ${R}_{i}\in \{M\to D,D\to D,M\to M,D\to M\}$. There are many meta-path types as shown in Fig. 8. For example, one meta-path type ${P}_{4}=M\to D\to M\to D$ is a *3-Length* (*3-L* for short) meta-path type.

Second, given a meta-path type *P*, there may be multiple paths following it, which are called meta-path instances. For example, as shown in Fig. 8, one meta-path instance of $P=M\to M\to D\to D$ is $p={r}_{2}\to {r}_{5}\to {d}_{3}\to {d}_{2}$. Here, ${r}_{i}$ and ${d}_{j}$ are the *i*-th miRNA and the *j*-th disease.

Third, meta-path based neighbour is a node linked to the target node with one meta-path instance, which helps to understand the target node. In a meta-path instance, we regard the first node as the target node and the last node as its meta-path based neighbour. For the meta-path instance $p$ in Fig. 8, the target node of $p$ is ${r}_{2}$. The meta-path based neighbour of ${r}_{2}$ in $p$ is ${d}_{2}$. It can be seen that, for the target node ${r}_{2}$, there are many neighbours based on the meta-path type $P$, which may have many instances.

Finally, we extract all meta-path instances from the miRNA-disease heterogeneous network.

### Linear transformations of MiRNAs and diseases

We modelled original features of miRNAs and diseases from miRNA similarity matrix $SM$ and disease similarity matrix $SD$, respectively. We obtained the *i*-th row in $SM$ as the feature of the *i*-th miRNA. Similarly, the *j*-th row in $SD$ was regarded as the feature of the *j*-th disease. We had to project the original features of miRNAs and diseases into the same latent vector space with linear transformations, as their dimensions are different.

For a miRNA *r*, we mapped the original features into the unified latent space as follows:

11 $${{\varvec{h}}}_{r}={{\varvec{W}}}^{R}\cdot {{\varvec{x}}}_{r}$$

here ${{\varvec{h}}}_{r}\in {R}^{z}$ is the transformed latent vector of miRNA *r*, and ${{\varvec{x}}}_{r}\in {R}^{{d}_{r}}$ is the original feature of miRNA $r$. ${{\varvec{W}}}^{R}\in {R}^{{z\times d}_{r}}$ is the linear transformation matrix for miRNAs, which is a learnable parameter.

In the same way, the original feature of disease *d* is mapped into the unified latent space as follows*:*

12 $${{\varvec{h}}}_{d}={{\varvec{W}}}^{D}\cdot {{\varvec{x}}}_{d}$$

here ${{\varvec{h}}}_{d}\in {R}^{z}$ is the transformed latent vector of disease *d*, and ${{\varvec{x}}}_{d}\in {R}^{{d}_{d}}$ is the original feature of disease *d*, ${{\varvec{W}}}^{D}\in {R}^{{z\times d}_{d}}$ is the linear transformation matrix for diseases. ${{\varvec{W}}}^{D}$ is a learnable parameter.

In Fig. 7, the nodes with shadow are the transformed representations of original miRNAs and diseases.

### The mean encoder of MiRNAs and diseases based on a single meta-path instance

Given a meta-path instance $p$, for a fixed target $u$ (the circle node with a shadow in Fig. 7) after the transformation, its measurable features are implied in the sequences of $p$. Therefore, structural and semantic information of $u$ can be gained from $p$. We let $v$ be the neighbour of $u$ ($u$ is a miRNA or disease) in a single meta-path instance $p$. The relative information between $u$ and $v$ is implied in $p$. To obtain this information, we used a mean encoder, which takes the mean of all the node vectors in $p$, to transform node sequence in $p$ to a single vector as follows:

13 $${{\varvec{h}}}_{u}^{p}=MEAN\left(\sum_{t{\in M}^{p}}{{\varvec{h}}}_{t}^{p}\right)$$

here ${{\varvec{h}}}_{u}^{p}{\in R}^{z}$ is the transformed vector of the node sequence of $p$. ${M}^{p}$ indicates nodes set in the sequence of $p$, which includes $u$ and $v$. ${{\varvec{h}}}_{u}^{p}$ is the latent vector of node $u$ embedded by a single meta path instance $p$.

### Attention-aware meta-path type encoder of MiRNAs and diseases

For a fixed target $u$, in the sight of a meta-path type $P$, there were many meta-path instances with different neighbours. For instance, there are two meta-path instances,${r}_{2}\to {r}_{5}\to {d}_{3}\to {d}_{2}$ and ${r}_{2}\to {r}_{1}\to {d}_{2}\to {d}_{1}$, for miRNA ${r}_{2}$ as shown in Fig. 8. The relative information implied in the two meta-path instances is not equal. To integrate all information of various meta-path instances with the same meta-path type and distinguish their importance to represent the target node $u$, we aggregated them into a single vector with graph attention.

14 $${e}_{u}^{p}=ReLU\left({{\varvec{a}}{\varvec{t}}{\varvec{t}}}_{p}\cdot {[{\varvec{h}}}_{u}||{{\varvec{h}}}_{u}^{p}]\right)$$

15 $${e{^{\prime}}}_{u}^{p}=\frac{\mathrm{exp}\left({e}_{u}^{p}\right)}{\sum_{q\in P}\mathrm{exp}\left({e}_{u}^{q}\right)}$$

16 $${{\varvec{h}}}_{u}^{P}=sigmoid\left(\sum_{p\in P}{e{^{\prime}}}_{u}^{p}\cdot {{\varvec{h}}}_{u}^{p}\right)$$

here ${{\varvec{a}}{\varvec{t}}{\varvec{t}}}_{p}{\in R}^{2z}$ is the attention parameter for meta-path instance $p$, $||$ is the vector concatenation, ${e}_{u}^{p}$ indicates the contribution of meta-path instance $p$ to target $u$, and ${e{^{\prime}}}_{u}^{p}$ is the normalization of ${e}_{u}^{p}$ by using the softmax function among all possible neighbours of $u$ based on meta-path type $P$. For all $p\in P$ with a target $u$, the comprehensive representation of $u$ can be gained by the weighted sum of all meta-path instances as shown in Formula (16).

### Attention-aware fusion of multiple meta-path types

Suppose there are $N$ meta-paths types in miRNA-disease heterogeneous networks, we defined a set of meta-path types ${\mathbb{P}}=\{{P}_{1},{P}_{2},\ldots ,{P}_{N}\}$. The representation of a target $u$ by different meta-path types can be defined as ${{\varvec{h}}}_{u}^{{P}_{i}}{\in R}^{z},i\in [1,N]$. Considering the distinct contribution of different meta-path types because of different lengths and patterns, we also employed attention mechanisms to get the final representation of $u$.

17 $${{w}^{{\prime}}}_{u}^{{P}_{i}}=ReLU\left({{\varvec{a}}{\varvec{t}}{\varvec{t}}}_{{P}_{i}}\cdot {{\varvec{h}}}_{u}^{{P}_{i}}\right)$$

18 $${w}_{u}^{{P}_{i}}=\frac{\mathrm{exp}\left({{w}^{{\prime}}}_{u}^{{P}_{i}}\right)}{\sum_{{P}_{i}\in {\mathbb{P}}}\mathrm{exp}\left({{w}^{{\prime}}}_{u}^{{P}_{i}}\right)}$$

19 $${{\varvec{h}}}_{u}^{\mathbb{P}}=\sum_{{P}_{i}\in {\mathbb{P}}}{{w}^{{\prime}}}_{u}^{{P}_{i}}\cdot {{\varvec{h}}}_{u}^{{P}_{i}}$$

here ${{\varvec{a}}{\varvec{t}}{\varvec{t}}}_{{P}_{i}}{\in R}^{z}$ is the attention parameter for meta-path type ${P}_{i}$. Moreover, ${w}_{u}^{{P}_{i}}$ indicates the contribution of meta-path type ${P}_{i}$ to target $u$. ${w{^{\prime}}}_{u}^{{P}_{i}}$ is the normalization of ${w}_{u}^{{P}_{i}}$ by using the softmax function among all meta-path types. Therefore, ${{\varvec{h}}}_{u}^{\mathbb{P}}{\in R}^{z}$ stands for the node representation fused by all meta-path types with meta-path type attention.

Up to now, the representation of a miRNA or disease with underlying information in meta-paths was modelled by the above three encoders.

### Predicting MiRNA-disease associations with model training

After fulfilling the steps introduced above, we obtained ${{\varvec{h}}}_{u}^{\mathbb{P}}$ as the final representation of a miRNA or disease, which includes the global information in miRNA-disease interactions. To achieve representations that are as correct as possible, we need to train the parameters of graph embedding, such as ${{\varvec{W}}}^{R}{,{{\varvec{W}}}^{D},{\varvec{a}}{\varvec{t}}{\varvec{t}}}_{p}$ and ${{\varvec{a}}{\varvec{t}}{\varvec{t}}}_{{P}_{i}}$, with mini-batch learning. According to our data, the main aim of training our model is to make the distance between two nodes which have a connection in the miRNA-disease heterogeneous network as small as possible. This means that the parameters of our model can be learned by minimizing the following loss function:

20 $$Loss=\sum_{\left(u,v\right)\in \mathcal{P}}\mathrm{log}\,sigmoid\left(dist\left(u,v\right)\right) -\sum_{\left(u,v\right)\in \mathcal{N}}\mathrm{log}\,sigmoid\left(dist\left(u,v\right)\right)$$

where $\mathcal{P}$ is the set of positive node pairs with approved relationships or high similarity and $\mathcal{N}$ is the negative node pairs with unknown relationships or low similarity. $dist\left(\cdot \right)$ is the similarity measurement by the Manhattan distance of two nodes.

21 $$dist\left(u,v\right)=\sum_{i=1}^{z}\left|{u}_{i}-{v}_{i}\right|.$$

## Supplementary information

**Additional file 1.** Supplementary tables for case studies..

## Data Availability

The data sets and source code that support the findings of this study are available in https://github.com/dangdangzhang/M2GMDA. A web service for M2GMDA is available at https://132.232.17.50:8080/M2GMDA.jsp.

## Abbreviations

MiRNAs: Micro ribonucleic acids
RWR: Random walk with restart
SVM: Support vector machine
SDNE: Structural deep network embedding
LOOCV: Leave one out cross validation
AUC: Area under the curve
ROC: Receiver operating characteristics
M2GMDA: Multiple meta-paths fusion graph embedding model to predict the unknown MiRNA-disease associations
