# Supplementary material for: Predicting MiRNA-disease associations by multiple meta-paths fusion graph embedding model
Source: BMC Bioinformatics. 2020 Oct 21;21:470. doi: 10.1186/s12859-020-03765-2 (PMC7579830; doi:10.1186/s12859-020-03765-2)
Supplement: Supplementary file 1 — Additional file 1. Supplementary tables for case studies.. [file 12859_2020_3765_MOESM1_ESM.docx]

**Supplementary Tables for Case Studies**

Table S1. Top 50 miRNAs associated with prostate neoplasms

| miRNA |  | Evidence |  | miRNA | Evidence |
| --- | --- | --- | --- | --- | --- |
| hsa-mir-21 |  | dbDEMC, PhenomiR |  | hsa-let-7b | dbDEMC, PhenomiR |
| hsa-mir-155 |  | dbDEMC, PhenomiR |  | hsa-mir-199a | dbDEMC, PhenomiR |
| hsa-mir-146a |  | dbDEMC, PhenomiR |  | hsa-mir-34c | Unconfirmed |
| hsa-mir-17 |  | dbDEMC, PhenomiR |  | hsa-mir-210 | dbDEMC, PhenomiR |
| hsa-mir-145 |  | dbDEMC, PhenomiR |  | hsa-mir-181a | dbDEMC |
| hsa-mir-20a |  | dbDEMC, PhenomiR |  | hsa-mir-15a | dbDEMC, PhenomiR |
| hsa-mir-34a |  | dbDEMC, PhenomiR |  | hsa-let-7c | dbDEMC |
| hsa-mir-125b |  | dbDEMC |  | hsa-mir-200a | dbDEMC, PhenomiR |
| hsa-mir-126 |  | dbDEMC, PhenomiR |  | hsa-mir-182 | dbDEMC, PhenomiR |
| hsa-mir-221 |  | dbDEMC, PhenomiR |  | hsa-mir-146b | PhenomiR |
| hsa-mir-92a |  | dbDEMC, PhenomiR |  | hsa-mir-29c | dbDEMC, PhenomiR |
| hsa-mir-16 |  | dbDEMC, PhenomiR |  | hsa-mir-9 | PhenomiR |
| hsa-mir-18a |  | dbDEMC, PhenomiR |  | hsa-mir-181b | dbDEMC, PhenomiR |
| hsa-let-7a |  | dbDEMC, PhenomiR |  | hsa-let-7d | dbDEMC, PhenomiR |
| hsa-mir-143 |  | dbDEMC, PhenomiR |  | hsa-mir-142 | PhenomiR |
| hsa-mir-200c |  | dbDEMC, PhenomiR |  | hsa-mir-34b | dbDEMC, PhenomiR |
| hsa-mir-19b |  | dbDEMC, PhenomiR |  | hsa-mir-218 | dbDEMC, PhenomiR |
| hsa-mir-1 |  | dbDEMC, PhenomiR |  | hsa-mir-133b | dbDEMC, PhenomiR |
| hsa-mir-200b |  | dbDEMC, PhenomiR |  | hsa-let-7e | dbDEMC, PhenomiR |
| hsa-mir-19a |  | dbDEMC, PhenomiR |  | hsa-mir-148a | dbDEMC, PhenomiR |
| hsa-mir-29a |  | dbDEMC, PhenomiR |  | hsa-mir-27a | dbDEMC, PhenomiR |
| hsa-mir-31 |  | dbDEMC, PhenomiR |  | hsa-mir-203 | dbDEMC, PhenomiR |
| hsa-mir-222 |  | dbDEMC, PhenomiR |  | hsa-mir-10b | dbDEMC, PhenomiR |
| hsa-mir-29b |  | dbDEMC, PhenomiR |  | hsa-mir-30a | PhenomiR |
| hsa-mir-223 |  | dbDEMC, PhenomiR |  | hsa-mir-205 | dbDEMC, PhenomiR |

Table S2. The top 50 miRNAs associated with pancreatic neoplasms

| miRNA | Evidence | miRNA | Evidence |
| --- | --- | --- | --- |
| hsa-mir-21 | dbDEMC, PhenomiR | hsa-mir-223 | dbDEMC, PhenomiR |
| hsa-mir-155 | dbDEMC, PhenomiR | hsa-let-7b | dbDEMC, PhenomiR |
| hsa-mir-146a | dbDEMC, PhenomiR | hsa-mir-199a | dbDEMC, PhenomiR |
| hsa-mir-17 | dbDEMC, PhenomiR | hsa-mir-210 | dbDEMC, PhenomiR |
| hsa-mir-145 | dbDEMC, PhenomiR | hsa-mir-34c | PhenomiR |
| hsa-mir-20a | dbDEMC, PhenomiR | hsa-mir-15a | dbDEMC, PhenomiR |
| hsa-mir-34a | dbDEMC, PhenomiR | hsa-let-7c | dbDEMC |
| hsa-mir-125b | dbDEMC, PhenomiR | hsa-mir-29c | dbDEMC, PhenomiR |
| hsa-mir-126 | dbDEMC, PhenomiR | hsa-mir-9 | dbDEMC, PhenomiR |
| hsa-mir-221 | dbDEMC, PhenomiR | hsa-mir-200a | dbDEMC, PhenomiR |
| hsa-mir-92a | dbDEMC, PhenomiR | hsa-mir-146b | dbDEMC |
| hsa-mir-16 | dbDEMC, PhenomiR | hsa-mir-182 | dbDEMC, PhenomiR |
| hsa-mir-18a | dbDEMC, PhenomiR | hsa-mir-181b | dbDEMC, PhenomiR |
| hsa-let-7a | dbDEMC, PhenomiR | hsa-let-7d | dbDEMC, PhenomiR |
| hsa-mir-19b | dbDEMC, PhenomiR | hsa-mir-30a | dbDEMC, PhenomiR |
| hsa-mir-1 | dbDEMC, PhenomiR | hsa-mir-142 | PhenomiR |
| hsa-mir-143 | dbDEMC, PhenomiR | hsa-mir-106b | dbDEMC, PhenomiR |
| hsa-mir-19a | dbDEMC, PhenomiR | hsa-mir-218 | dbDEMC, PhenomiR |
| hsa-mir-29a | dbDEMC, PhenomiR | hsa-let-7e | dbDEMC, PhenomiR |
| hsa-mir-200c | dbDEMC, PhenomiR | hsa-mir-133b | dbDEMC, PhenomiR |
| hsa-mir-200b | dbDEMC, PhenomiR | hsa-mir-34b | dbDEMC, PhenomiR |
| hsa-mir-31 | dbDEMC, PhenomiR | hsa-mir-205 | PhenomiR |
| hsa-mir-222 | dbDEMC, PhenomiR | hsa-mir-7 | dbDEMC, PhenomiR |
| hsa-mir-181a | dbDEMC, PhenomiR | hsa-mir-148a | dbDEMC, PhenomiR |
| hsa-mir-29b | dbDEMC, PhenomiR | hsa-mir-195 | dbDEMC, PhenomiR |

Table S3. Cases results for more new diseases (lymphoma, lung neoplasms, colorectal neoplasms, breast neoplasms)

| Disease | The predicted top 50 miRNAs | Unconfirmed miRNAs |
| --- | --- | --- |
| lymphoma | hsa-mir-21,hsa-mir-155,hsa-mir-17,hsa-mir-146a,hsa-mir-20a,hsa-mir-34a,hsa-mir-125b,hsa-mir-145,hsa-mir-221,hsa-mir-126,hsa-mir-92a,hsa-mir-18a,hsa-mir-16,hsa-let-7a,hsa-mir-19b,hsa-mir-143,hsa-mir-19a,hsa-mir-29a,hsa-mir-1,hsa-mir-200c,hsa-mir-222,hsa-mir-31,hsa-mir-29b,hsa-mir-200b,hsa-mir-223,hsa-let-7b,hsa-mir-181a,hsa-mir-199a,hsa-mir-15a,hsa-mir-34c,hsa-mir-210,hsa-let-7c,hsa-mir-182,hsa-mir-146b,hsa-mir-181b,hsa-mir-9,hsa-mir-142,hsa-mir-200a,hsa-let-7d,hsa-mir-29c,hsa-mir-34b,hsa-let-7e,hsa-mir-30a,hsa-mir-148a,hsa-mir-106b,hsa-mir-10b,hsa-mir-218,hsa-mir-27a,hsa-mir-133b,hsa-mir-205 | hsa-mir-34c  hsa-mir-142 |
| lung neoplasms | hsa-mir-21,hsa-mir-155,hsa-mir-146a,hsa-mir-17,hsa-mir-145,hsa-mir-34a,hsa-mir-20a,hsa-mir-125b,hsa-mir-126,hsa-mir-221,hsa-mir-16,hsa-mir-92a,hsa-mir-18a,hsa-let-7a,hsa-mir-143,,hsa-mir-19b,hsa-mir-200c,hsa-mir-1,hsa-mir-19a,hsa-mir-200b,hsa-mir-29a,hsa-mir-31,hsa-mir-222,hsa-mir-29b,hsa-mir-15a,hsa-mir-223,hsa-let-7b,hsa-mir-199a,hsa-mir-181a,hsa-mir-210,hsa-mir-34c,hsa-let-7c,hsa-mir-182,hsa-mir-200a,hsa-mir-146b,hsa-mir-9,hsa-mir-29c,hsa-mir-181b,hsa-let-7d,hsa-mir-142,hsa-mir-106b,hsa-mir-218,hsa-mir-34b,hsa-mir-133b,hsa-let-7e,hsa-mir-195,hsa-mir-148a,hsa-mir-27a,hsa-mir-30a,hsa-mir-10b | hsa-mir-142 |
| colorectal neoplasms | hsa-mir-21,,hsa-mir-155,hsa-mir-146a,hsa-mir-17,hsa-mir-145,hsa-mir-20a,hsa-mir-34a,hsa-mir-125b,hsa-mir-126,hsa-mir-221,hsa-mir-92a,hsa-mir-16,hsa-mir-18a,hsa-let-7a,hsa-mir-143,hsa-mir-19b,hsa-mir-200c,hsa-mir-1,hsa-mir-19a,hsa-mir-29a,hsa-mir-223,hsa-mir-29b,hsa-mir-200b,hsa-mir-31,hsa-mir-222,hsa-mir-15a,hsa-let-7b,hsa-mir-199a,hsa-mir-181a,hsa-mir-210,hsa-mir-34c,hsa-mir-29c,hsa-let-7c,hsa-let-7d,,hsa-mir-200a,hsa-mir-182,hsa-mir-146b,hsa-mir-9hsa-mir-181b,hsa-mir-142,hsa-mir-106b,hsa-let-7e,hsa-mir-205,hsa-mir-218,hsa-mir-133b,hsa-mir-34b,hsa-mir-101,hsa-let-7f,hsa-mir-214,hsa-mir-100 | None |
| breast neoplasms | hsa-mir-21,hsa-mir-155,hsa-mir-146a,hsa-mir-17,hsa-mir-145,hsa-mir-20a,hsa-mir-34a,hsa-mir-125b,hsa-mir-126,hsa-mir-221,hsa-mir-92a,hsa-mir-16,hsa-mir-18a,hsa-let-7a,hsa-mir-143,hsa-mir-19b,hsa-mir-200c,hsa-mir-1,hsa-mir-19a,hsa-mir-200b,hsa-mir-29a,hsa-mir-31,hsa-mir-222,hsa-mir-29b,hsa-mir-223,hsa-let-7b,hsa-mir-199a,hsa-mir-181a,hsa-mir-210,hsa-mir-34c,hsa-mir-15a,hsa-mir-142,hsa-let-7c,hsa-mir-200a,hsa-mir-146b,hsa-mir-182,hsa-mir-9,hsa-mir-181b,hsa-mir-29c,hsa-let-7d,hsa-mir-218,hsa-mir-150,hsa-let-7e,hsa-mir-34b,hsa-mir-133b,hsa-mir-148a,hsa-mir-10b,hsa-mir-27a,hsa-mir-30a,hsa-mir-203 | hsa-mir-146b |

Table S4. The top 50 miRNAs associated with colorectal neoplasms

| miRNA | Evidence | miRNA | Evidence |
| --- | --- | --- | --- |
| hsa-let-7d | dbDEMC, PhenomiR | hsa-mir-451 | dbDEMC, HMDD |
| hsa-let-7f | dbDEMC, PhenomiR | hsa-mir-192 | dbDEMC, PhenomiR, HMDD |
| hsa-mir-125a | dbDEMC, PhenomiR, HMDD | hsa-mir-15a | dbDEMC, PhenomiR, HMDD |
| hsa-let-7i | dbDEMC, PhenomiR, HMDD | hsa-mir-98 | dbDEMC, PhenomiR |
| hsa-mir-106b | dbDEMC, PhenomiR, HMDD | hsa-mir-29c | dbDEMC, PhenomiR, HMDD |
| hsa-mir-132 | dbDEMC, PhenomiR, HMDD | hsa-mir-32 | dbDEMC, PhenomiR, HMDD |
| hsa-mir-29b | dbDEMC, PhenomiR, HMDD | hsa-mir-204 | dbDEMC, PhenomiR, HMDD |
| hsa-mir-223 | dbDEMC, PhenomiR, HMDD | hsa-mir-153 | dbDEMC, HMDD |
| hsa-mir-30c | dbDEMC, PhenomiR, HMDD | hsa-mir-488 | dbDEMC |
| hsa-let-7g | dbDEMC, PhenomiR, HMDD | hsa-mir-302d | dbDEMC |
| hsa-mir-191 | dbDEMC, PhenomiR, HMDD | hsa-mir-99b | dbDEMC, PhenomiR, HMDD |
| hsa-mir-205 | HMDD | hsa-mir-100 | dbDEMC, PhenomiR, HMDD |
| hsa-mir-214 | dbDEMC, PhenomiR, HMDD | hsa-mir-23b | dbDEMC, PhenomiR, HMDD |
| hsa-mir-30d | dbDEMC, PhenomiR, HMDD | hsa-mir-383 | dbDEMC, HMDD |
| hsa-mir-101 | dbDEMC, PhenomiR, HMDD | hsa-mir-15b | dbDEMC, PhenomiR |
| hsa-mir-20b | dbDEMC, PhenomiR, HMDD | hsa-mir-130b | dbDEMC, PhenomiR, HMDD |
| hsa-mir-30e | dbDEMC, PhenomiR, HMDD | hsa-mir-196b | dbDEMC, HMDD |
| hsa-mir-30b | dbDEMC, PhenomiR, HMDD | hsa-mir-299 | PhenomiR |
| hsa-mir-151 | dbDEMC, PhenomiR | hsa-mir-10a | dbDEMC, PhenomiR |
| hsa-mir-302b | dbDEMC | hsa-mir-198 | dbDEMC, PhenomiR, HMDD |
| hsa-mir-302c | dbDEMC, HMDD | hsa-mir-193b | dbDEMC, HMDD |
| hsa-mir-24 | dbDEMC, PhenomiR, HMDD | hsa-mir-452 | dbDEMC, HMDD |
| hsa-mir-296 | PhenomiR | hsa-mir-424 | dbDEMC, PhenomiR, HMDD |
| hsa-mir-302a | dbDEMC, HMDD | hsa-mir-212 | dbDEMC, PhenomiR |
| hsa-mir-520b | dbDEMC | hsa-mir-449b | HMDD |
